# Effects of flavonoid-induced oxidative stress on anti-H5N1 influenza a virus activity exerted by baicalein and biochanin A

**DOI:** 10.1186/1756-0500-7-384

**Published:** 2014-06-23

**Authors:** Martin Michaelis, Patchima Sithisarn, Jindrich Cinatl Jr

**Affiliations:** 1 Institute for Medical Virology, Clinics of the Goethe-University, Paul Ehrlich-Str. 40, 60596 Frankfurt am Main, Germany; 2 Current address: Centre for Molecular Processing and School of Biosciences, University of Kent, Canterbury CT2 7NJ, UK; 3 Current address: Faculty of Veterinary Medicine, Kasetsart University, Bangkok 10900, Thailand

**Keywords:** H5N1, Biochanin A, Baicalein, Antiviral, Reactive oxygen species, N-acetyl-L-cysteine

## Abstract

**Background:**

Different flavonoids are known to interfere with influenza A virus replication. Recently, we showed that the structurally similar flavonoids baicalein and biochanin A inhibit highly pathogenic avian H5N1 influenza A virus replication by different mechanisms in A549 lung cells. Here, we investigated the effects of both compounds on H5N1-induced reactive oxygen species (ROS) formation and the role of ROS formation during H5N1 replication.

**Findings:**

Baicalein and biochanin A enhanced H5N1-induced ROS formation in A549 cells and primary human monocyte-derived macrophages. Suppression of ROS formation induced by baicalein and biochanin A using the antioxidant N-acetyl-L-cysteine strongly increased the anti-H5N1 activity of both compounds in A549 cells but not in macrophages.

**Conclusions:**

These findings emphasise that flavonoids induce complex pharmacological actions some of which may interfere with H5N1 replication while others may support H5N1 replication. A more detailed understanding of these actions and the underlying structure-activity relationships is needed to design agents with optimised anti-H5N1 activity.

## Findings

Highly pathogenic influenza A viruses including H5N1 viruses represent a major pandemic threat. Complication rates are much higher in H5N1 patients than in seasonal influenza or pandemic H1N1/09 patients [1-4]. As of 24^th^ January 2014, 650 confirmed human H5N1 cases had resulted in 386 deaths (http://www.who.int).

During an initial pandemic phase, matched vaccines will be restricted and antiviral drugs will be critical. The efficacy of the approved anti-influenza drugs (adamantanes, neuraminidase inhibitors) is limited, resistant strains emerge, and H5N1 strains appear to be less sensitive to the established anti-influenza drugs than seasonal influenza strains [1,4-13]. Hence, additional anti-influenza therapies are needed.

**Figure 1:**
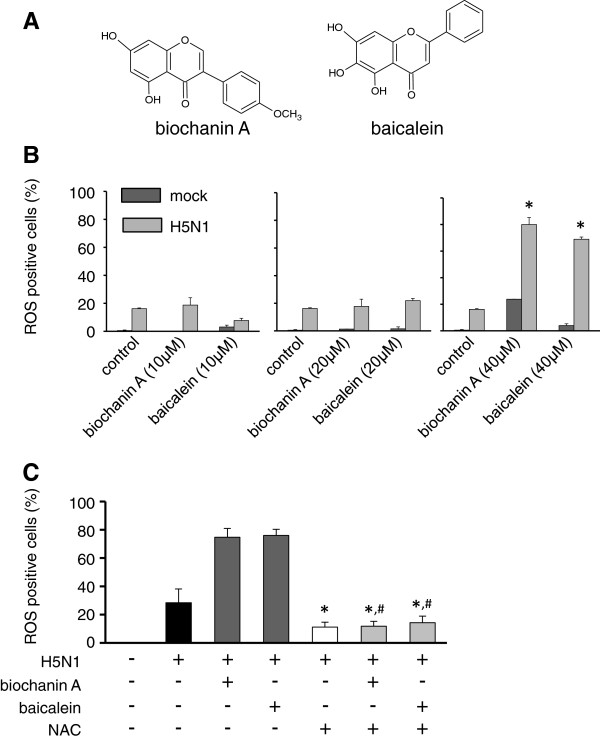
**Effects of baicalein and biochanin on reactive oxygen species (ROS) formation in A549 cells infected with H5N1 strain A/Thailand/1(Kan-1)/04.** A549 cells were treated continuously with the investigated flavonoids and/ or N-acetyl-L-cysteine (NAC) starting with a 1 h pre-incubation period prior to infection with H5N1 strain A/Thailand/1(Kan-1)/04 (MOI 0.01). ROS formation was detected at 24 h post infection. **A)** Chemical structures of biochanin A and baicalein; **B)** Effects of different baicalein or biochanin A concentrations on H5N1-induced ROS formation. *P < 0.05 relative to virus control; **C)** Effects of N-acetyl-L-cysteine (NAC) 5 mM on baicalein 40 μM- or biochanin A 40 μM-induced ROS formation in H5N1-infected A549 cells. ‘-’ indicates absence of virus or respective compound, ‘+’ indicates presence of virus or respective compound. *P < 0.05 relative to virus control, ^#^P < 0.05 relative to respective flavonoid alone. Values are presented as mean ± S.D. from three different independent experiments.

In 2009, the “WHO public health research agenda for Influenza” expressed a need for additional drugs including those that exert immunomodulatory effects and recommended to investigate natural products for anti-influenza activity (http://www.who.int). Flavonoids are known to exert multiple pharmacological effects including anti-inflammatory and anti-viral activities including inhibition of seasonal influenza A (H1N1) viruses [14-19]. They may interfere with the influenza virus neuraminidase [19-21], the virus host cell uptake [20,22], or cellular signalling events like the activation of nuclear factor kB (NFkB), AKT, ERK 1/2, p38, and/or JNK [23-27]. We showed recently that the flavonoids biochanin A and baicalein interfere with H5N1 replication in lung epithelial cells but that only baicalein inhibited H5N1 replication in primary human monocyte-derived macrophages [28]. Although biochanin A and baicalein are closely related structures (Figure 1A), they differed in their antiviral mechanisms. Inhibition of the H5N1 neuraminidase appeared to substantially contribute to the anti-H5N1 effects exerted by baicalein but not by biochanin A. Biochanin A interfered in contrast to baicalein with H5N1-induced activation of constituents of cellular signalling pathways [28] that are known to be involved in influenza virus replication such as AKT, ERK 1/2, and NFκB [10,29-32]. Notably, the effects of baicalein and biochanin A on H5N1 replication are complex and additional antiviral mechanisms are likely to contribute to their anti-H5N1 activities.

Flavonoids are known to differ in their effects on the formation of reactive oxygen species (ROS). They may display anti- or pro-oxidative effects [33]. Influenza virus replication is influenced by the cellular redox status [34]. The inhibition of virus-induced ROS formation by different strategies including the use of the antioxidant N-acetyl-L-cysteine (NAC) was shown to inhibit influenza A virus replication including H5N1 strains [34-36]. Here, we investigated the effects of baicalein and biochanin A on H5N1-induced ROS formation and the combined effects of baicalein and biochanin A in combination with the antioxidant NAC on H5N1 replication.

A549 cells (human lung carcinoma; ATCC, Manassass, VA, USA: CCL-185) and Vero cells (African green monkey kidney; ATCC: CCL81) were cultivated as described previously [28]. Human monocytes were isolated from buffy coats of healthy donors (Institute of Transfusion Medicine and Immune Haematology, German Red Cross Blood Donor Centre, Goethe-University, Frankfurt/Main, Germany) and CD14^+^ monocytes were differentiated into MDMs as described previously [28]. Cells were infected with H5N1 strain A/Thailand/1(Kan-1)/04 (obtained from Dr. Puthavathana, Mahidol University, Bangkok, Thailand) and virus titres were determined as 50% tissue culture infectious dose (TCID_50_/mL) as described previously [28]. Flavonoids, NAC, or their combinations were present starting from a 1 h pre-incubation period prior to infection. For the identification of statistically significant differences (P < 0.05), two groups were compared by Student’s *t*-test, more groups by ANOVA followed by subsequent stepwise multiple comparison procedure using the Student-Newman-Keuls method.

**Figure 2:**
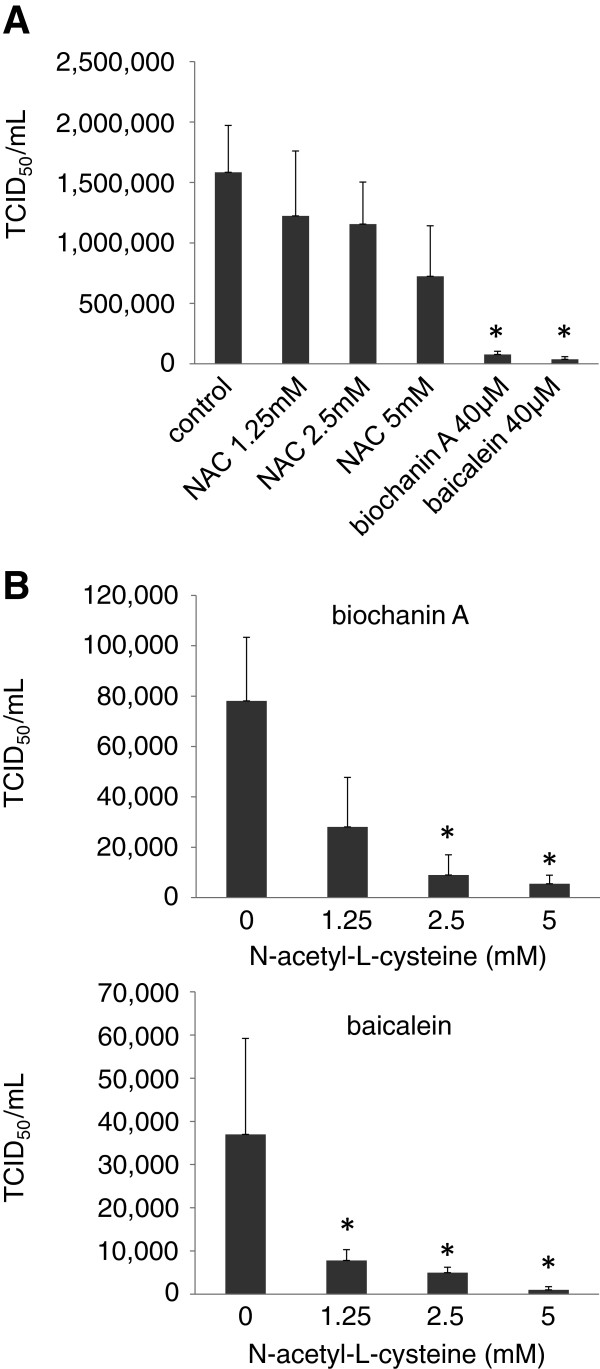
**Effects of baicalein and biochanin A on H5N1 titres in combination with N-acetyl-L-cysteine (NAC).** A549 cells were infected with H5N1 strain A/Thailand/1(Kan-1)/04 (MOI 0.01). Drugs were continuously present starting with a 1 h pre-incubation period. Virus titres were determined 48 h post infection. **A)** Effects of NAC on H5N1 replication, *P < 0.05 relative to virus control; **B)** Effects of NAC on H5N1 titres in the presence of the flavonoids baicalein 40 μM or biochanin A 40 μM, *P < 0.05 relative to flavonoid alone. Values are presented as mean ± S.D. from three different independent experiments.

**Figure 3:**
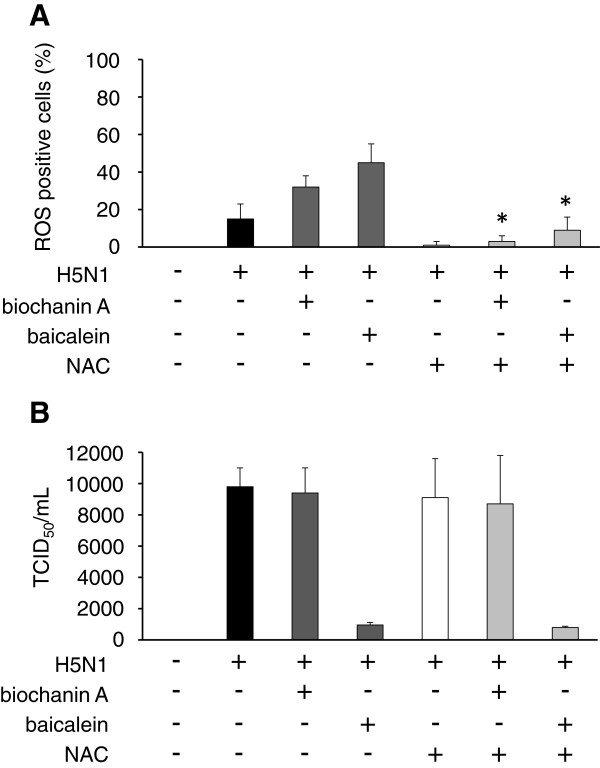
**Effects of baicalein and biochanin A on reactive oxygen species (ROS) formation and H5N1 replication in primary human monocyte-derived macrophages in combination with N-acetyl-L-cysteine (NAC).** Macrophages were infected with H5N1 strain A/Thailand/1(Kan-1)/04 (MOI 1). Drugs were continuously present starting with a 1 h pre-incubation period. Virus titres were determined 48 h post infection. **A)** Effects of flavonoids 40 μM and/or NAC 5 mM on H5N1-induced ROS formation, ‘-’ indicates absence of virus or respective compound, ‘+’ indicates presence of virus or respective compound. *P < 0.05 relative to flavonoids alone; **B)** Effects of baicalein 40 μM or biochanin A 40 μM in the presence or absence of NAC 5 mM on H5N1 titres. ‘-’ indicates absence of virus or respective compound, ‘+’ indicates presence of virus or respective compound. Values are presented as mean ± S.D. from three different independent experiments.

H5N1 infection of A549 cells at a multiplicity of infection (MOI) 0.01 resulted in enhanced ROS formation compared to control 24 h after infection (Figure 1B) as indicated by the use of the Image-iT LIVE Green Reactive Oxygen Species Kit (Molecular Probes, distributed by Invitrogen, Karlsruhe, Germany). Baicalein and biochanin A (both obtained from Indofine Chemical Company, Hillsborough, NJ, USA) did not influence ROS levels in non-infected or H5N1-infected cells in concentrations up to 20 μM. However, at a concentration of 40 μM both compounds increased the ROS levels in non-infected as well as H5N1-infected cells (Figure 1B) despite the differences in their modes of anti-H5N1 action [28]. An NAC (obtained from Alexis, distributed by Axxora, Germany, dissolved in unsupplemented MEM and adjusted to pH 7.4 with NaOH) concentration of 5 mM was sufficient to reduce the ROS levels below the levels observed in non-treated H5N1-infected A549 cells (Figure 1C).Next, we investigated whether the reduction of baicalein- or biochanin A-induced enhanced ROS levels in H5N1-infected A549 cells by NAC influences the antiviral effects of these flavonoids. H5N1 (MOI 0.01)-infected A549 cells were treated with baicalein 40 μM or biochanin A 40 μM in combination with NAC in concentrations ranging from 1.25 to 5 mM. NAC did not affect cell viability alone or in combination with baicalein or biochanin A in the investigated concentrations as indicated by the CellTiter-Glo® Luminescent Cell Viability Assay (Promega GmbH, Mannheim, Germany) (data not shown). While NAC 5 mM alone moderately reduced H5N1 titres (2.2-fold reduction), NAC 2.5 mM or 1.25 mM did not significantly affect virus titres (Figure 2A). However, NAC reduced H5N1 titres in combination with baicalein or biochanin A in a dose-dependent manner in this concentration range in A549 cells (Figure 2B). Notably, NAC also inhibited baicalein- and biochanin A-induced oxidative stress in H5N1-infected primary human monocyte-derived macrophages but did not affect H5N1 replication in this cell type (Figure 3). Human monocytes had been isolated from buffy coats of healthy donors, obtained from the Institute of Transfusion Medicine and Immune Haematology, German Red Cross Blood Donor Center, Johann Wolfgang Goethe-University, Frankfurt am Main.

In conclusion, we show that two flavonoids that interfere with H5N1 replication by different mechanisms of action exert similar effects at the level of ROS induction. Baicalein interferes with the H5N1 neuraminidase activity but biochanin does not. Biochanin A (but not baicalein) inhibits the activation of signalling molecules involved in H5N1-induced signalling including AKT, ERK 1/2, and NFκB [28]. Despite these differences in their anti-H5N1 mechanisms, both compounds enhanced H5N1-induced ROS formation in A549 cells, and the efficacy of both compounds was enhanced by the antioxidant NAC. In contrast, inhibition of flavonoid-induced ROS formation by NAC did not affect virus replication in H5N1-infected macrophages. These findings emphasise that flavonoids, a class of natural compounds known to exert anti-influenza effects [16-19,28], induce a complex range of pharmacological actions by which they modify influenza A virus replication including highly pathogenic avian H5N1 strains. These actions may be cell type-specific and include pro- and antiviral effects. The overall activity may be the result of the totality of effects exerted by a certain flavonoid in a certain cell type. A more detailed understanding of these actions and the underlying structure-activity relationships is needed in order to design structures with optimised anti-influenza activity.

## Abbreviations

NAC: N-acetyl-L-cysteine; ROS: Reactive oxygen species.

## Competing interests

The authors declare that they have no competing interests.

## Authors’ contributions

MM and JC designed the study, analysed the data, and wrote the manuscript. PS performed experiments and analysed data. All authors read and approved the final manuscript.
